# Aromatherapy: Does It Help to Relieve Pain, Depression, Anxiety, and Stress in Community-Dwelling Older Persons?

**DOI:** 10.1155/2014/430195

**Published:** 2014-07-13

**Authors:** Shuk Kwan Tang, M. Y. Mimi Tse

**Affiliations:** ^1^Department of Orthopaedics & Traumatology, United Christian Hospital, Kowloon, Hong Kong; ^2^School of Nursing, The Hong Kong Polytechnic University, Kowloon, Hong Kong

## Abstract

To examine the effectiveness of an aromatherapy programme for older persons with chronic pain. The community-dwelling elderly people who participated in this study underwent a four-week aromatherapy programme or were assigned to the control group, which did not receive any interventions. Their levels of pain, depression, anxiety, and stress were collected at the baseline and at the postintervention assessment after the conclusion of the four-week programme. Eighty-two participants took part in the study. Forty-four participants (37 females, 7 males) were in the intervention group and 38 participants (30 females, 8 males) were in the control group. The pain scores were 4.75 (SD 2.32) on a 10-point scale for the intervention group and 5.24 (SD 2.14) for the control group before the programme. There was a slight reduction in the pain score of the intervention group. No significant differences were found in the same-group and between-group comparisons for the baseline and postintervention assessments. The depression, anxiety, and stress scores for the intervention group before the programme were 11.18 (SD 6.18), 9.64 (SD 7.05), and 12.91 (SD 7.70), respectively. A significant reduction in negative emotions was found in the intervention group (*P* < 0.05). The aromatherapy programme can be an effective tool to reduce pain, depression, anxiety, and stress levels among community-dwelling older adults.

## 1. Introduction

Pain is a global and common problem among older persons worldwide. In Hong Kong, the older population is increasing in proportion to the population as a whole, from 13% in 2011 to 30% in 2041 [1]. The annual growth rate of older persons from 1991 to 1996 was 5.1% [2]. It is anticipated that the burden on health services and social welfare will be heavier as a result of the ageing population in Hong Kong.

Pain brings many problems to older people, including physical and psychological dysfunctions. The prevalence of pain in community-dwelling older persons is high, ranging from 25% to 50% [3]. Older persons have been found to suffer from different levels of pain [4–6]. Most suffer from pain originating from the musculoskeletal system [4, 5, 7, 8]. The pain score rated on a 10-point scale by the older persons was 4.6 to 7.5 and described as moderate to severe [4, 5, 7].

Pain is the total suffering of a person in the physical, psychological, social, and spiritual aspects, according to Saunders' widely accepted total pain concept [9]. This concept helps to guide health professionals to view pain using a multidimensional and holistic approach. Pain can cause problems to an individual in a single physical, psychological, social, and spiritual aspect, or in two or more interrelated aspects. Physical pain leads to psychological distress and social interruption, affecting relationships with family, relatives, and friends. It also induces a fear that the condition of pain will further deteriorate. Depression, anxiety, stress, and functional status have been found in different studies to be associated with chronic pain [10–16]. Studies have reported that when older persons have persistent pain, the prevalence of depression is high and anxiety develops in relation to the repetition of pain-inducing activities. Stress was found to mediate the pain disability of patients with lower back pain [17]. With the presence of pain, the mobility level of older persons declines, particularly as pain levels increase [11].

Pharmacological and nonpharmacological interventions have been effective in managing pain in older persons. Older persons use analgesics as their pharmacological approach [4, 18]. However, physicians may be reluctant to prescribe adequate analgesics because they might not have had sufficient training in this area, and therefore tend to prescribe medications on an “as needed” (PRN) basis or upon request [19, 20]. Older persons also tend to wait until the pain cannot be tolerated before asking for the PRN analgesics [21]. They also fear the adverse effects brought about by analgesics [22].

Older persons without pain education place a lower priority on nonpharmacological interventions for managing pain [4]. They tend to administer pain relief strategies by themselves [23]. Therefore, a pain education programme can help older persons to relieve pain-related distress and improve pain management [8].

Aromatherapy using aromatic plants to treat medical and health problems has a long history in western society [24]. Specifically, aromatherapy involves the use of essential oils to restore balance and improve well-being. A holistic approach is applied in aromatherapy, which treats the person as a whole to strengthen his/her immune system in order to fight against diseases [25, 26]. There are different methods of administering essential oils, including topical application, inhalation, baths, and compresses. Research using aromatherapy as the intervention has demonstrated its effectiveness in reducing pain in adults and infants [27, 28].

Pain and the olfactory pathways in humans have been found to be related. In a gene study involving the gene* SCN9A*, a loss of function of the gene led to a loss of function in pain sensation and odour perception [29].

**Figure 1:**
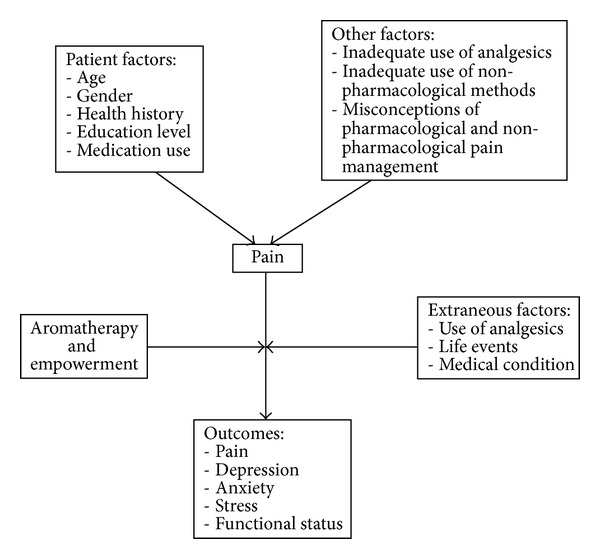
Conceptual framework of the present study.

Studies have been conducted using positron emission tomography (PET) to examine the effect of inhaling an odour on the reduction of pain. The results showed that with the inhalation of a pleasant odour, pain intensity was reduced in human subjects [30–32]. Laboratory- and community-based studies produced similar findings, namely, that pain in adults and older persons can be reduced with the inhalation of odours and essential oils [33–37] (see Figure 1).

*Aim of the Study*. The aim of the present study was to examine the effectiveness of a four-week aromatherapy programme for older persons with chronic pain, as well as their levels of depression, anxiety, and stress.

## 2. Method

### 2.1. Design and Sample

This was a quasi-experimental pretest and posttest control group study. The size of the sample was calculated using Cohen's d table. Based on a previous study on pain and aromatherapy in Hong Kong, the following parameters were set: effect size 0.8, power 0.9, and 5% alpha [34].

Ethical approval was granted by the Human Subjects Ethics Subcommittee of the Hong Kong Polytechnic University. Older persons were recruited from local community elderly centres. The participants were aged 65 or above, members of their community elderly centres, able to understand and communicate in Cantonese and able to follow instructions, and had chronic pain for at least 3 months before the commencement of the study. They were required to pass the Abbreviated Mental Test and the olfactory test. Older persons who were allergic to essential oils or perfumes or had terminal illnesses or a history of diseases affecting the olfactory senses were excluded.

Eighty-two participants were recruited for the present study. Thirty-eight were assigned to the control group and 44 to the intervention group.

### 2.2. Intervention

#### 2.2.1. Centre-Based Sessions

An aromatherapist was consulted on the content of the aromatherapy programme, which was a tailor-made four-week programme consisting of four centre-based sessions and self-administered home-based sessions. The centre-based sessions were held once per week in community elderly centres. Knowledge on pain, pain in older persons, and aromatherapy was introduced during the centre-based sessions. In the sessions, lavender and bergamot essential oils were administered by inhalation.

#### 2.2.2. Self-Administered Home-Based Aromatherapy

The self-administered home-based aromatherapy programme was designed to enable the participants to continue practising aromatherapy at home. Each participant was given a bottle of aromatic spray to carry out the self-administered home-based aromatherapy. The content of the aromatic spray was designed by the aromatherapist to be suitable for use by the elderly. The aromatic spray was made with diluted lavender and bergamot essential oils and lavender hydrolats. The ratio of the concentration of the lavender essential oils to the bergamot essential oils to the lavender hydrolats was 2 : 1 : 2.5, as suggested by the aromatherapist. During the centre-based sessions, the participants were shown how to use the aromatic spray at home. A demonstration and a return demonstration were carried out to ensure that the participants knew how to use the aromatic spray correctly. The aromatic spray was used externally, by inhalation (see Table 1).

### 2.3. Procedure

The study took place at community elderly centres in a local area. The community elderly centres were similar. A baseline assessment before the intervention was conducted using a questionnaire to collect data on the demographics of the participants and their pain, depression, anxiety, and stress levels. The Geriatric Pain Assessment was adopted for assessing pain [38]. The questionnaire included questions on pain measured using a 10-point scale, factors that might alleviate and exacerbate the pain, and any activities that needed to be avoided in relation to the pain. The Depression, Anxiety, and Stress Scale (DASS-21) was used to measure the negative emotional status of depression, anxiety, and stress [39]. A total of 21 items were included in the questionnaire, with seven items in each subscale of depression, anxiety, and stress. The negative emotional status of depression, anxiety, and stress were graded as follows: normal, mild, moderate, severe, and very severe. A postintervention assessment was conducted after the conclusion of the four-week aromatherapy programme.

### 2.4. Data Analysis

The Statistical Package for Social Science, SPSS for Windows version 17.0, was used for the quantitative data analysis. *P* < 0.05 was considered the level of statistical significance. The Chi-square test was used to measure the demographic data of the control and intervention groups. The dependent variables were pain, depression, anxiety, and stress. The Wilcoxon's signed ranks test was used to examine the dependent variables and compare the baseline data to the postintervention data. The Mann-Whitney *U* Test was used for the dependent variables when comparing the control and intervention groups.

## 3. Result

There were 44 participants in the intervention group (37 females and 7 males) and 38 in the control group (30 females and 8 males), for a total of 82 participants in the study.

### 3.1. Demographic Data: Intervention Group versus Control Group

Of the participants, 27.3% and 34.2% were aged over 76 to 80 in the intervention and control groups, respectively. There was no significant difference in gender, age, marital status, education level, personal health history, living status, financial status, and previous occupation (*P* > 0.05) (see Table 2).

### 3.2. Pain Scores and Pain Sites: Baseline and Post-Intervention Assessments

All of the participants from both groups had had chronic pain for more than 3 months. They had different patterns and frequency of pain. No significant difference was found when comparing the pain situations of the two older groups at the baseline and postintervention assessments (*P* > 0.05) (see Table 3).

At the baseline, the pain score of the intervention group was 4.75 (SD 2.32) and that of the control group was 5.24 (SD 2.14). After the aromatherapy programme, the pain score of the intervention group had decreased to 4.66 (SD 2.56). In the control group, the pain score was 4.79 (SD 2.19) at the postintervention assessment. No significant difference was found in the between-group and within-group comparisons (see Table 4).

**Figure 2:**
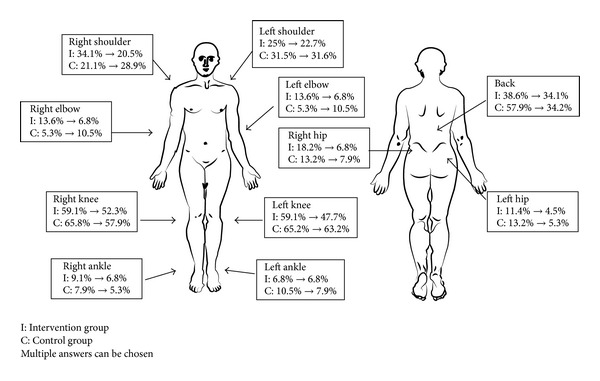
Location of pain in intervention and control groups.

The most common sites of pain for the older persons at the baseline and postintervention assessments were the knees and back.  Figure 2 shows the percentage of participants who suffered from pain in each particular body part.

### 3.3. The Use of Pharmacological Interventions: Baseline and Postintervention Assessments

Significant differences in the use of analgesics were found in both groups when comparing the baseline and postintervention assessments within the same group (*P* < 0.05). There was no significant difference between the groups (*P* > 0.05). There was an increase in the use of analgesics in the intervention group after the aromatherapy programme, while in the control group there was a decrease in use.

Panadol was the analgesic most commonly used by participants in the intervention and control groups. There was a significant difference in the types of analgesics used in the intervention group when comparing the baseline and postintervention assessments (*P* < 0.05). A significant difference in frequency (prescription of PRN) was also found in the within-group comparison, but not in the between-group comparison.

### 3.4. The Use of Nonpharmacological Interventions: Baseline and Postintervention Assessments

There was increased use of nonpharmacological interventions in both groups after the aromatherapy programme. In the intervention group, the percentage of participants using nonpharmacological interventions increased from 84.1% to 100%. As shown in Table 5, no participants in the intervention group reported that they had any reason for not using or knowing about nonpharmacological interventions after the aromatherapy programme. Analgesic balm or oils, massage, and hot pads were the top three choices of nonpharmacological interventions adopted by both groups at the baseline and postintervention assessments. A significant difference in the participants' perceptions of the effectiveness of nonpharmacological interventions was shown in the intervention group (*P* < 0.05) (see Table 5).

### 3.5. Depression, Anxiety, and Stress Level: Baseline and Postintervention Assessments

The baseline and postintervention assessment results for depression, anxiety, and stress in the intervention group are shown in Table 6. When the baseline and postintervention assessments were compared, the intervention group showed decreased scores for depression, anxiety, and stress. Significant differences in the depression, anxiety, and stress scores were found at the postintervention assessment when comparing the intervention and control groups (*P* < 0.05). Significant differences were noted in the depression, anxiety, and stress scores in the intervention group when comparing the baseline and postintervention assessment results (*P* < 0.05) (see Table 6).

## 4. Discussion

The present study shows that the aromatherapy programme was effective in reducing the pain, depression, anxiety, and stress levels of older persons in the intervention group. Under the total pain concept, a person not only suffers from physical pain but also from psychological distress [9]. The fact that pain can induce psychological distress in older persons is consistent with the total pain concept. As illustrated by Saunders [9], psychological distress can be related to progressive pain. Although in the present study a decrease in pain scores was noted in both groups, psychological distress increased to a greater degree in the control group than in the intervention group. This can be related to the effect of the aromatherapy programme, which provided the intervention group with adequate information on pain and pain management. Older persons in the control group did not receive this information and were still uncertain as to their pain and pain management, resulting in an increased level of psychological distress. The findings were consistent with those of previous studies, namely, that the inadequate pain management of older persons results in decreased enjoyment of life, and pain management programmes can decrease pain-related distress [4, 8].

Laboratory-based studies have proven that the inhalation of odours or essential oils is effective at reducing pain under the use of PET [31, 32]. The reduction in pain scores in the present study was minimal. Pain scores ranging from four to five were found in both groups, indicating mild to moderate pain. The pain in older persons originated in the musculoskeletal system, while in laboratory-based studies the pain was induced by thermodes, hot water, or cold water. The nature of the pain was different. In the laboratory-based studies, the essential oils were inhaled rather than topically applied to pain sites as in the community-based studies [34, 35]. The method of administering the essential oils and the duration of the aromatherapy were factors affecting the impact of the aromatherapy programme in pain management.

Lavender and bergamot essential oils are antidepressants and relaxants [25, 40]. Essential oils can be absorbed by inhalation into the olfactory pathway and from there to the brain [41]. The scores on depression, anxiety, and stress decreased in the intervention group after the aromatherapy programme, but there was increased psychological distress in the control group. The results were consistent with those of previous studies, namely, that aromatherapy was able to relieve negative emotional symptoms [25, 40, 42].

Pleasant odours can induce a positive mood in a person [25]. Essential oils administered in the centre-based sessions and aromatic spray used in the self-administered home-based sessions exposed the older persons to pleasant aromas. Their mood was lifted after inhaling the essential oils and aromatic spray, resulting in decreased depression, anxiety, and stress scores.

Aromatherapy has been used to treat diseases for decades in western countries [24]. Using aromatherapy as a method of managing pain was a new concept to the older Chinese persons in the present study, as none in either group were familiar with aromatherapy at the baseline assessment. To introduce them to new nonpharmacological interventions, they were given more exposure and choices for managing pain. The fact that the pain management programme was able to empower older persons with knowledge about pain and the necessary skills to manage their health problems is consistent with the findings of previous studies [8, 43]. The aromatherapy programme consisted of centre-based sessions and self-administered home-based aromatherapy. Accepting and cooperating with the aromatherapy programme, the older persons in the intervention group benefited from decreased levels of pain, depression, anxiety, and stress after completing the programme. The positive effect of the programme was also supported by the increased use of aromatherapy and aromatic spray by the participants in the intervention group after the completion of the programme.

The present study has limitations in that the samples were recruited by convenience and only older persons were included. The results cannot be applied to other populations. The duration of the sessions may not have been enough to generate a sustained effect in the older persons in the management of their pain. It is recommended that, in future studies, a third group of participants be added as placebo to prevent the occurrence of the Hawthorn effect.

## 5. Conclusion

As the proportion of older persons in the population increases, pain will undoubtedly become an even greater problem in the future. Pain is inevitable in the face of age and decreased physical functions. Under the total pain concept espoused by Saunders [9], pain is multidimensional, affecting people in their physical, psychological, social, and spiritual aspects. Psychological well-being is also affected when an older person suffers from chronic pain. Pharmacological and nonpharmacological interventions have been found to be effective in managing chronic pain in older persons. Nonpharmacological interventions are considered to have fewer side-effects and tend to be preferred by older persons. According to the total pain concept, there is a close relationship between pain and psychological distress.

A four-week aromatherapy programme was designed and implemented as a nonpharmacological intervention for managing pain in older persons. Depression, anxiety, and stress levels were significantly reduced in the intervention group after the aromatherapy programme, showing that aromatherapy can help to maintain the psychological health of community-dwelling older persons.

Keeping older persons pain-free and in good psychological health is the key to healthy ageing. Educational programmes can help to promote knowledge about pain and pain management in older persons.

## Figures and Tables

**Table 1:** Interventions of the aromatherapy programme.

Week	Teaching content (40 minutes)	Activity (20 minutes)	Self-administered aromatherapy
1	(i) Introduction to pain: mechanism, assessment, effects of pain on physical and psychological health, pharmacological and nonpharmacological approaches (ii) Aromatherapy: introduction, history, and indications (iii) Deep breathing exercises: introduction and theory	Practical session on deep breathing exercises and aromatherapy	(i) Carried out at home by older persons (ii) Aromatic spray given with education on use and precautions
2	Introducing different types of essential oils and uses, indications for usage	Practical session on deep breathing exercises and aromatherapy	
3	Demonstration of how to make an aromatherapy toolbox	(i) Odour testing game (ii) Practical session on deep breathing exercises and aromatherapy	
4	Making an aroma decoration—a towel rabbit	(i) Practical session on deep breathing exercises and aromatherapy (ii) Drinking of fruit tea	

**Table 2:** Demographic data of the intervention and control groups.

	Intervention group (*n* = 44)	Control group (*n* = 38)	Group difference
	*n* (%) Mean ± SD	*n* (%) Mean ± SD	*P* value^#^
Gender			0.627
Male	7 (15.9)	8 (21.1)	
Female	37 (64.1)	30 (78.9)	
Age			0.074
65–70	9 (20.5)	6 (15.8)	
71–75	8 (18.2)	7 (18.4)	
76–80	12 (27.3)	13 (34.2)	
Over 80	15 (34.1)	12 (31.6)	
Marital status			0.298
Single	3 (6.8)	2 (5.3)	
Married	18 (40.9)	17 (44.7)	
Divorced	1 (2.3)	0 (0)	
Widowed	22 (50)	19 (50)	
Education Level			0.559
No formal education	16 (36.4)	19 (50)	
Primary level	23 (52.3)	13 (34.2)	
Secondary level	5 (11.4)	5 (13.2)	
Tertiary level	0 (0)	1 (2.6)	
Personal health history (multiple answers can be chosen)			
No chronic illnesses	6 (13.6)	3 (7.9)	0.536
Hypertension	31 (70.5)	26 (68.4)	0.130
Diabetes mellitus	10 (22.7)	13 (34.2)	0.825
Heart diseases	4 (9.1)	6 (15.8)	0.593
Stroke	5 (11.4)	1 (2.6)	0.693
Gout	6 (13.6)	12 (31.6)	0.392
Respiratory diseases	4 (9.1)	6 (15.8)	0.435
Arthritis	8 (18.2)	13 (34.2)	0.145
Cataract	13 (29.5)	11 (28.9)	0.715
Others	11 (25)	11 (28.9)	0.932
Living Status			0.485
Alone	16 (36.4)	23 (60.5)	
With spouse	9 (20.5)	3 (7.9)	
With spouse and children	10 (22.7)	6 (15.8)	
With children	8 (18.2)	5 (13.2)	
With relatives or friends	1 (2.3)	1 (2.6)	
Financial status			0.881
Very poor	0 (0)	0 (0)	
Poor	4 (9.1)	5 (13.2)	
Average	31 (70.5)	21 (55.3)	
Good	9 (20.5)	12 (31.6)	
Very good	0 (0)	0 (0)	
Previous occupation			0.729
Worker	19 (42.2)	20 (52.6)	
Clerk	1 (2.3)	1 (2.6)	
Specialized	17 (38.8)	15 (39.5)	
Housework	7 (15.9)	2 (5.3)	

Percentages may not add up to 100% because of rounding.

^#^The Chi-square test was used.

*A *P* value of <0.05 was considered statistically significant.

**Table 3:** Geriatric pain assessment of the intervention and control groups.

	Intervention group (*n* = 44)			Control group (*n* = 38)			Group difference	
	Baseline	Postintervention	*P* value^#^	Baseline	Postintervention	*P* value^#^	Baseline	Postintervention
	*n* (%)	*n* (%)		*n* (%)	*n* (%)		*P* value^#^	*P* value^#^
Pain more than 3 months			—			—	—	—
Yes	44 (100)	44 (100)		38 (100)	38 (100)			
No	0 (0)	0 (0)		0 (0)	0 (0)			
Pattern of pain			0.086			0.180	0.299	0.575
Occasional	24 (54.5)	28 (63.6)		18 (47.4)	21 (55.3)			
Persistent	20 (45.5)	16 (36.4)		20 (52.6)	17 (44.7)			
Frequency (per day)			0.059			0.070	0.384	0.667
1	3 (6.8)	7 (15.9)		3 (7.9)	3 (7.9)			
2	5 (11.4)	11 (25)		3 (7.9)	7 (18.4)			
3	11 (25)	3 (6.8)		5 (13.2)	6 (15.8)			
4	3 (6.8)	1 (2.3)		6 (15.8)	1 (2.6)			
5	1 (2.3)	2 (4.5)		2 (5.3)	2 (5.3)			
More than 5	21 (47.7)	20 (45.5)		19 (50)	19 (50)			

Percentages may not add up to 100% because of rounding.

^#^The Chi-square test was used.

*A *P* value of <0.05 was considered statistically significant.

**Table 4:** Pain scores of the intervention and control groups.

	Intervention group (*n* = 44)				Control group (*n* = 38)				Group difference			
	Baseline	Postintervention	Z^a^	P value^a^	Baseline	Postintervention	Z^a^	P value^a^	Baseline		Postintervention	
	Mean ± SD	Mean ± SD			Mean ± SD	Mean ± SD			U^b^	P value^b^	U^b^	P value^b^
Pain score	4.75 ± 2.32	4.66 ± 2.56	0.22	0.830	5.24 ± 2.14	4.79 ± 2.19	1.16	0.247	721	0.274	789	0.654

^a^The Wilcoxon signed ranks test was used to compare the baseline data to the postintervention data of the control and intervention groups.

^b^The Mann-Whitney *U* Test was used to compare the control and intervention groups.

*A *P* value of <0.05 was considered statistically significant.

**Table 5:** Use of pharmacological and nonpharmacological interventions by the intervention and control groups.

	Intervention group (*n* = 44)			Control group (*n* = 38)			Group difference	
	Baseline	Postintervention	*P* value^#^	Baseline	Postintervention	*P* value^#^	Baseline	Postintervention
	*n* (%)	*n* (%)		*n* (%)	*n* (%)		*P* value^#^	*P* value^#^
Use of analgesics	16 (36.4)	17 (38.6)	0.000∗	19 (50)	18 (47.4)	0.001∗	0.087	0.272
Types of analgesics used (multiple answers can be chosen)							0.513	0.714
Panadol	13 (29.5)	14 (31.8)	0.000∗	12 (31.6)	15 (39.5)	0.088		
Tramadol	1 (2.3)	3 (6.8)		2 (5.3)	1 (2.6)			
Others	2 (4.5)	0 (0)		5 (13.2)	2 (5.3)			
Frequency (prescription on PRN)			0.000∗			0.010∗	0.629	0.767
Once daily	3 (6.8)	5 (11.4)		7 (18.4)	7 (18.4)			
Twice daily	4 (9.1)	6 (13.6)		5 (13.2)	4 (10.5)			
TDS	4 (9.1)	3 (6.8)		5 (13.2)	2 (5.3)			
QID	5 (11.4)	2 (4.5)		0 (0)	5 (13.2)			
Q4H	0 (0)	0 (0)		1 (2.6)	0 (0)			
Q6H	1 (2.3)	0 (0)		1 (2.6)	0 (0)			
Use of nonpharmacological interventions	37 (84.1)	44 (100)	—	28 (73.7)	37 (97.4)	0.090	0.731	—
Reasons for not using nonpharmacological interventions (multiple answers can be chosen)								
Do not know enough about nonpharmacological interventions	4 (9.1)	0 (0)	—	6 (15.8)	0 (0)	—	—	—
No medical/nursing staff have introduced such interventions	2 (4.5)	0 (0)	—	3 (7.9)	1 (2.6)	—	—	—
Do not believe this is an effective method of pain relief	2 (4.5)	0 (0)	—	2 (5.3)	0 (0)	—	—	—
No nursing staff to carry out the interventions	1 (2.3)	0 (0)	—	1 (2.6)	0 (0)	—	—	—
No facilities to carry out the interventions	1 (2.3)	0 (0)	—	1 (2.6)	0 (0)	—	—	—
Types of nonpharmacological interventions used (multiple answers can be chosen)								
Aromatherapy	0 (0)	37 (84.1)	—	0 (0)	0 (0)	—	—	—
Analgesic balm/oil	31 (70.5)	34 (77.3)	—	24 (63.2)	32 (84.2)	—	—	—
Massage	14 (31.8)	17 (38.6)	—	7 (18.4)	9 (23.7)	—	—	—
Hot pad	2 (4.5)	0 (0)	—	11 (28.9)	0 (0)	—	—	—
Resting in bed	2 (4.5)	0 (0)	—	7 (18.4)	2 (5.3)	—	—	—
Watching television	2 (4.5)	0 (0)	—	6 (15.8)	0 (0)	—	—	—
Cold pad	1 (2.3)	0 (0)	—	3 (7.9)	0 (0)	—	—	—
Chatting	1 (2.3)	0 (0)	—	2 (5.3)	0 (0)	—	—	—
Listening to music	0 (0)	1 (2.3)	—	1 (2.6)	0 (0)	—	—	—
Mediation	0 (0)	0 (0)	—	0 (0)	0 (0)	—	—	—
Deep breathing	0 (0)	0 (0)	—	0 (0)	0 (0)	—	—	—
Others	14 (31.8)	0 (0)	—	4 (10.5)	6 (15.8)	—	—	—
Participants' perceptions of the effectiveness of nonpharmacological interventions	28 (63.6)	38 (86.4)	0.008∗	22 (57.9)	35 (92.1)	0.296	0.380	0.168

Percentages may not add up to 100% because of rounding.

^#^The Chi-square test was used.

*A *P* value of <0.05 was considered statistically significant.

**Table 6:** Depression Anxiety and Stress Scales of the intervention and control groups.

	Intervention group (*n* = 44)				Control group (*n* = 38)				Group difference			
	Baseline	Postintervention	*Z* ^a^	*P* value^a^	Baseline	Postintervention	*Z* ^a^	*P* value^a^	Baseline		Postintervention	
	Mean ± SD	Mean ± SD			Mean ± SD	Mean ± SD			*U* ^b^	*P* value^b^	*U* ^b^	*P* value^b^
Depression	11.18 ±6.18	7.05 ± 5.11	3.43	0.001∗	12.11 ± 7.32	12.89 ± 6.70	1.14	0.256	834	0.985	404	0.000∗
Anxiety	9.64 ± 7.05	5.64 ± 3.72	3.49	0.000∗	8.26 ± 5.94	10.05 ± 5.47	1.87	0.062	745	0.395	423	0.000∗
Stress	12.91 ± 7.70	7.55 ± 6.37	3.38	0.001∗	11.37 ± 7.07	11.74 ± 7.63	0.31	0.761	709	0.233	574	0.014∗

^a^The Wilcoxon signed ranks test was used to compare the baseline data to the postintervention data of the control and intervention groups.

^b^The Mann-Whitney *U* Test was used to compare the control and intervention groups.

*A *P* value of <0.05 was considered statistically significant.
